# Influence of temperature on the torsional properties of two thermally treated NiTi rotary instruments

**DOI:** 10.1590/0103-6440202305094

**Published:** 2023-03-06

**Authors:** Roberto Barreto Osaki, Clovis Monteiro Bramante, Rodrigo Ricci Vivan, Murilo Priori Alcalde, Pedro Henrique Souza Calefi, Marco Antonio Hungaro Duarte

**Affiliations:** 1 Department of Dentistry, Endodontics and Dental Materials, Bauru Dental School, University of São Paulo, Bauru, SP, Brazil.

**Keywords:** Nickel-titanium, torsional fatigue, body temperature, thermal treatment

## Abstract

This study aimed to evaluate the influence of temperature on torsional strength and angular deflection of two experimental NiTi rotary instruments manufactured from Blue and Gold thermal treatments and with identical cross-sections. A total of 40 experimental NiTi instruments 25.06 and with a triangular cross-section and manufactured from Blue and Gold thermal treatments were used (n=20). The torsional test was performed in the 3 mm from the tip of the instrument according to ISO 3630-1. The torsional test evaluated the torsional strength and angular deflection to failure at room temperature (21°C ± 1° C) and body temperature (36°C ±1°C). The fractured surface of each fragment was observed by using scanning electron microscopy (SEM). Data were analyzed using an unpaired t test for inter and intra-group comparison and the level of significance was set at 5%. The results showed that the body temperature did not affect the torsional strength and angular deflection of the instruments when compared with room temperature (P>0.05). However, at body temperature, the Blue NiTi instruments presented significantly lower angular deflection in comparison with Gold NiTi instruments (P<0.05). There was no significant difference regarding the torsional strength of the instruments at body temperature (P>0.05). The temperature did not affect the torsional strength of the instruments manufactured from Blue and Gold technology. However, the Blue NiTi instruments presented significantly lower angular deflection than Gold instruments at 36°C temperature.

## Introduction

The NiTi engine-driven instruments ensure safe and efficient root canal preparation of curved canals ^1^, ^2^. However, the risk of instrument separation continues to be a concernment for the clinicians due to this occurrence could affect the clinical outcome of endodontic treatment ^3^,^4^.

The instruments separation can occur by cyclic and torsional fatigue ^5^, ^6^. The cyclic fatigue occurs when the instruments are rotating inside a curved root canal and is submitted to continue force of compression and traction at the maximum point of the curvature ^3^,^6^. The torsional fatigue occurs during instruments rotation and the tip locks on the root canal walls ^3^,^6^. Regardless of the cause of instrument failure, the risk should be reduced ^3^,^4^.

The thermal treatment of the NiTi has been widely used for manufacturing of engine-driven NiTi instruments because tends to favor high flexibility, safer root canal preparation of curved canals, high angular deflection, and less canal transportation ^3^,^5^-^8^. However, the torsional strength to fracture tends to be reduced when compared to conventional NiTi ^6^. In addition, the design of the instrument (cross-section, taper, diameter of core has a strong influence on their mechanical properties of them ^2^,^3^,^4^,^8^,^9^.

Previous studies have shown that the cyclic fatigue resistance of the NiTi instruments is affected when exposed to body temperature ^11^,^13^-^15^. This fact could be explained because the thermal treatments present different temperatures of austenite-martensite transformation, affecting the flexibility at different temperatures ^6^. Klymus *et al* ^11^) reported that Blue and Gold thermal treatments presented a 50% and 19% in reduction on their cyclic fatigue resistance when exposed to body temperature, respectively. However, there is few information regarding the real impact of the temperature on the torsional properties of the NiTi instruments manufactured from different thermal treatments ^12^,^13^.

Silva *et al* ^12^ evaluated the torsional behavior of NiTi instruments manufactured from conventional and controlled memory technology with identical designs (tip diameter, taper, and cross-section). The authors concluded that the body temperature did not affect the torsional strength and angular deflection of the instruments. There are no reports that evaluated the torsional behavior of NiTi instruments with identical designs and manufactured different thermal treatments at body temperature. This evaluation would be important to ensure that the torsional properties of thermally treated NiTi instruments did not modify as occurred in the flexural properties. In addition, it would possible to select which thermally treated instrument would be safer for the root canal preparation of constricted canals.

The Blue and Gold technologies promotes good flexural properties for NiTi instruments ^7^. However, body temperature causes a negative impact on the cyclic fatigue ^7^,^12^,^13^. There are no reports evaluating the impact of the body temperature on the torsional strength and angular deflection of instruments manufactured by Blue and Gold technologies. Therefore, the aim of this study was to evaluate the impact of body temperature on the torsional strength and angular deflection of experimental NiTi instruments manufactured from Blue and Gold technologies and with identical designs (tip diameter, taper, and cross-section. The null hypothesis was as follow: ^1^ there is no significant difference in the torsional strength of the instruments at 21 °C and 36 °C, and 2 there is no significant difference in the angular deflection between the instruments at 21 °C and 36 °C temperature.

## Methods

Prior to the torsional tests, sample calculation was performed using G*Power v3.1 for Mac (Heinrich Heine, University of Düsseldorf) and selecting the Wilcoxon-Mann-Whitney test of the *t*-test family. An alpha-type error of 0.05, a beta power of 0.95, and an N2/N1 ratio of 1 were also stipulated. A total of ^(10)^ samples per group were indicated as the ideal size required for noting significant differences.

A total of 40 NiTi instruments representing the two experimental rotary instruments (*n* = 20 per system) were used in this study. The instruments presented identical geometric features (triangular cross-section design, taper 0.06 mm/mm, 0.25 mm of tip diameter, and 25mm long).

The experimental NiTi instruments were manufactured by Blue (EB) and Gold (EG) technologies (Mk Life, Porto Alegre, Brazil). The experimental Blue instruments present the same thermal treatment used to manufacture the X1 Blue File reciprocating instruments (Mk Life, Produtos Médicos e Odontológicos, Brazil). According to the manufacturer, the instruments are undergoing a complex heating-cooling proprietary process that results in a deposition of a Titanium Oxide layer on the surface of the NiTi, which creates a shape memory and flexible alloy. Also, the final austenitic temperature (Af) is to be around 38°C. The experimental Gold instruments present the same thermal treatment used to manufacture the Pro-T rotary instruments (Mk Life, Produtos Médicos e Odontológicos, Brazil). The manufacturer affirmed that the instruments are produced by a proprietary heating-cooling method that results in a visible gold-colored Titanium oxide layer, shape memory alloy, and austenitic final temperature around 50°C.

## Torsional fatigue test

Each instrument was inspected for defects or deformities before being tested under a stereomicroscope (Carls Zeiss, LLC, EUA) at 16x magnification; none of them were discarded.

The torsion tests were performed based on the International Organization for Standardization ISO 3630-^1^ (1992) specification using a torsion machine as previously described ^17^,^18^,^19^. A total of 40 instruments of each type (*n* = 20; 25 mm of length) were used to establish maximum torque torsional strength and angular deflection to failure under two experimental conditions, at room temperature (21°C ± 1°C) and body temperature (36°C ± 1°C).

**Figure 1:**
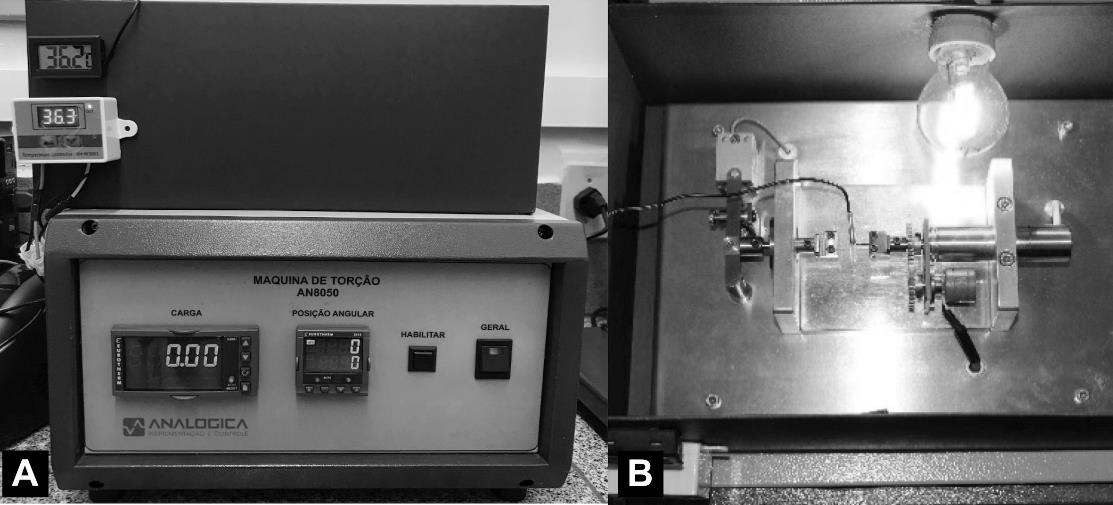
Representative image of the torsion machine test with the cabinet (A) and the inside view of the cabinet during torsional test (B).

The room temperature (21°C ± 1°C) was maintained by an air conditioner and monitored by a digital thermometer and one digital contact thermometer for the temperature of the instrument. For the torsional test at body temperature (36°C ± 1°C), a cabinet was mounted over the torsional machine device and incandescent light (100watt and 110V) was coupled for heating the cabinet (Figure 1). The temperature of the cabinet and of the instrument were also monitored. Then, when both temperatures were the same, the light was turned off and the torsional test was started.

The three-millimeter length of the instrument tip was clamped into a mandrel connected to a geared motor. The torque values were assessed by measuring the force exerted on a small load cell by a lever arm linked to the torsion axis. The geared motor operated in the clockwise direction at a speed set to 2 rpm. A specific software of the machine (MicroTorque; Analógica, Belo Horizonte, Brazil) recorded all data.

### SEM Evaluation

The fractured surfaces of all the instruments were examined by scanning electron microscopy (JEOL, JSM-TLLOA, Tokyo, Japan) to determine the topographic features of the fragments after torsional fatigue tests. Before SEM evaluation, the instruments were ultrasonically cleaned to remove debris. The fractured surfaces of the instruments were assessed at 200-x and at 1000-x magnification at the centers of the surfaces of the instruments.

### Statistical analysis

The data were first examined using the Kolmogorov-Smirnov test to analyze the normality of distribution. The results were analyzed using an unpaired t-test, and the level of significance was set at 5%. The Prism 6.0 software (GraphPad Software Inc., La Jolla, CA, USA) was used as the analytical tool.

## Results

### Torsional fatigue test

The mean and standard deviations of the torsional test at 21 °C and 36 °C temperature (torsional strength and angular deflection) are presented in Table 1. The results showed that the body temperature did not affect the torsional strength and angular deflection of the instruments when compared with room temperature (P>0.05). However, at body temperature the Blue NiTi instruments presented significantly lower angular deflection in comparison with Gold NiTi instruments (P<0.05). There was no significant difference regarding the torsional strength of the instruments at body temperature (P>0.05).

**Table 1:** Torque (N.cm) and angular deflection (°) of instruments tested.

Instruments	Torsional fatigue							
	Torque (N.cm)				Angles (°)			
	21°C		36°C		21°C		36°C	
	Mean	SD	Mean	SD	Mean	SD	Mean	SD
EB 25.06	1,867^a,A^	0,3445	1,683^a,A^	0,4622	389,6^a,A^	57,29	335,5^a,A^	42,69
EG 25.06	1,700^a,A^	0,3286	1,783^a,A^	0,5776	401,0^a,A^	51,66	393,7^a,B^	22,49

SD, standard deviation. Different superscript upper case letters in the same column indicate significant differences amongst groups (P < 0.05). Different superscript lower case letters in the same row indicate intragroup significant differences (P < 0.05).

### SEM Evaluation

**Figure 2:**
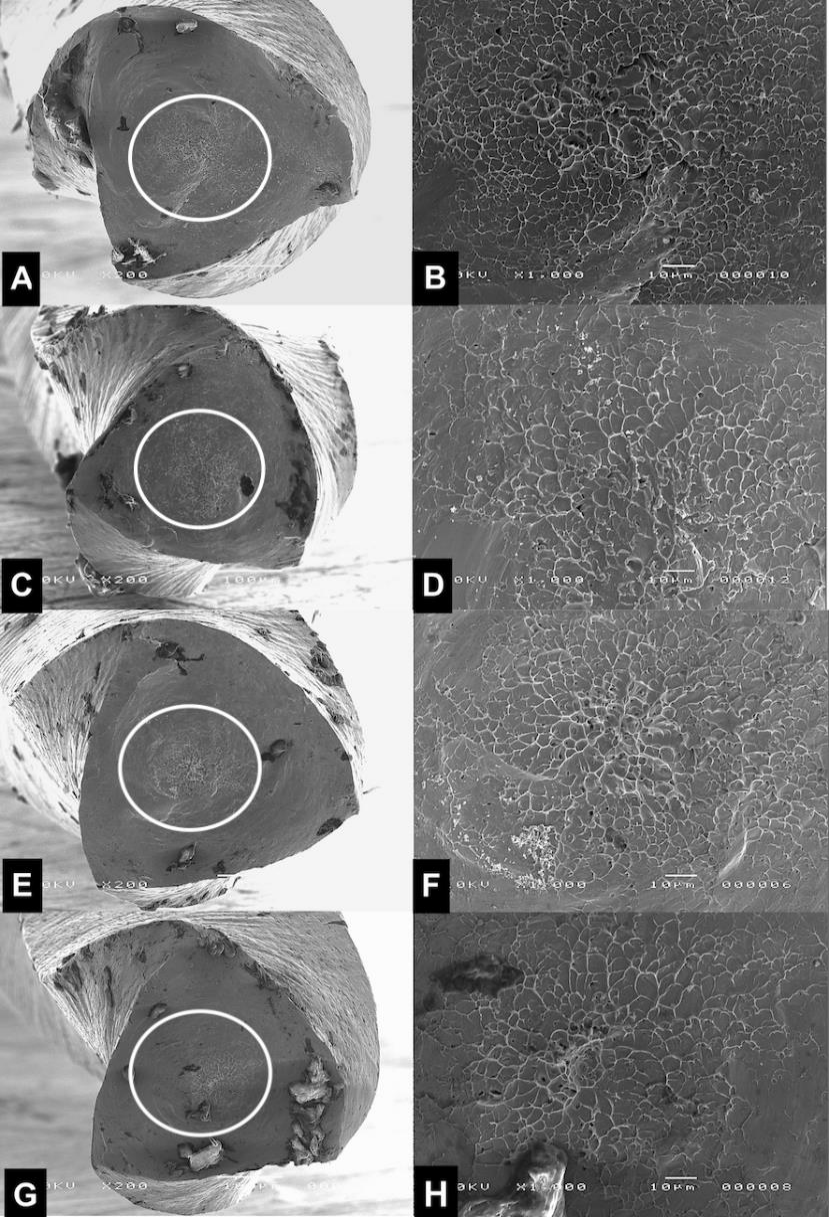
SEM images of fractured fragments of (A-B and E-F) Blue and (C-D and G-H) Gold experimental NiTi instruments at room temperature and body temperature, respectively. The first column shows the frontview images of the instruments at 200x magnification. The second column shows the multiple skewed dimples and concentric abrasion mark at 1000x magnification, showing the skewed dimples near the center of rotation are typical feature of torsional failure.

Scanning electron microscopy of the fragment surfaces showed similar and typical features of torsional failure for all instruments tested. All the instruments showed abrasion marks and fibrous dimples near the center of rotation (Figure 2).

## Discussion

The impact of temperature on the cyclic fatigue resistance of NiTi engine-driven instruments has been extensively evaluated ^12^,^14^ ,^16^. However, there is a few information regarding the effect of the temperature on the torsional properties of instruments manufactured from different thermal treatments, blue and gold. Therefore, the aim of this study was to evaluate the torsional properties of NiTi instruments with identical features (tip diameter, cross-section, and taper) manufactured from Gold and Blue technologies. Therefore, variables such as instrument tip size, taper, and cross-sectional design, that affect resistance to fracture by torsion were eliminated.

The torsional test was performed according to ISO 3630-1 specification, which was previously reported ^17^,^19^. There is only one study that evaluated the torsional resistance at body and room temperature ^12^, and this study used the same methodology with some modifications. This test aimed to simulate high torsional stress when the tip of the instrument lock in the root dentine during root canal preparation of constricted canal ^12^,^16^,^17^. It is important to highlight that this methodology did not represent the real condition inside the root canal. Also, this study aimed to demonstrate the effect of two different temperatures on the torsional strength and angular deflection in instruments manufactured by Blue and Gold technology. Therefore, future studies should be conducted to improve this method or simulate better clinical conditions. Despite this methodology did not used the instruments immersed in the water bath, the temperature of the instruments and the cabinet was totally controlled by digital thermometers during the test. In addition, the torsional test was performed fixing the 3 mm of the instruments and a continuous rotation of 2 RPM, which have no instrument friction, avoiding heating. This method was chosen due to the limitation of the device used, that does not allow instruments be tested immersed in a solution with controlled temperature. Other important information is that the only difference between the instruments is the thermal treatment. Therefore, variables such as instrument tip size, taper, cross-sectional design, that affects resistance to fracture by torsion was eliminated.

The first results of this study showed that there was no significant difference in the torsional properties of the instruments at 21 °C and 36 °C temperatures (P>0.05). Therefore, our first null hypothesis was accepted. The results of this study are in agreement with Silva *et al* ^12^, which reported that the temperature did not affect the torsional properties of instruments manufactured with conventional NiTi and controlled memory technology. Therefore, it could be suggested that the torsional properties of NiTi instruments manufactured by Blue and Gold technology are not affected by temperature.

The second part of this study compared (inter-group) the torsional strength and angular deflection between the Blue and Gold experimental instruments at 21 °C and 36 °C temperatures. The results showed there was a significant difference between the instruments manufactured from Gold technology regarding the angular deflection only at body temperature (P<0.05). Therefore, our second null hypothesis was partially rejected. Although previous studies reported that NiTi instruments manufactured with Blue technology presented greater flexibility than Gold treatment at body temperature ^11^,^18^,^19^, our results oppose it. These conflicting results could be explained mainly by differences in the different thermal treatments among the manufacturers and the type of environmental conditions (temperature-controlled ovens or immersed in different preheated solutions). Also, the results could be related with the temperature of austenite and martensite transformation. The manufacturer of the experimental instruments stated that the Af of Blue and Gold instruments are 38 °C and 50°C. Previous studies reported that the final temperature of austenite transformation (Af) of Blue technology ranges from 34.42- 38,5 °C, whereas Gold technology ranges from 50.1°C 6,9,11. Probably, the difference of Af Blue technology induced less percentage of martensite phase at body temperature, which affects the flexibility of the instruments and reduces the deformation capacity of the NiTi ^6^, ^11^,^13^,^15^.

The torsional properties of NiTi instruments should be considered during root canal preparation of constricted canal. There is no specific clinical indication of the Gold or Blue NiTi instruments on the literature. However, some authors reported that Gold and Blue NiTi instruments present less cyclic fatigue resistance when exposed at root canal temperature ^6^,^11^,^14^. Also, the higher Af temperature of Gold thermal treatment favor a less percentage reduction of cyclic fatigue than Blue, which is an important feature for root canal preparation of curved canal ^11^,^14^. Our results showed that both thermal treatments present the similar torsional strength at root canal temperature, which would ensure similar safety for torsional failure. However, the Gold thermal treatment present greater angular deflection at (36)°C. The higher angular deflection failure that an instrument can tolerate, the higher the elastic and plastic deformation before it breaks. This can be a safety feature because an occurrence of plastic deformation can be visualized and can be a warning that a torsional fracture is imminent ^3^,^9^,^16^. Therefore, based on your results, it could be suggested that a gold thermal treatment can be safer than blue during canal preparation of constricted root canal.

Future studies should be conducted using Differential Scanning Calorimetry (DSC) analysis to confirm the Af temperature reported by the manufacturer of the experimental instruments, which could assist to confirm our results. In addition, a clinical study could assess if there would be difference in the rate of fractured or deformed instruments between the both different thermal treatment during root canal preparation of constricted canals.

SEM analysis of the instruments fractured by torsion revealed fractured surface with morphologic characteristics of the ductile mode (multiple skewed dimples and circular abrasion marks near the center of rotation), which is in consonance with the results from several previous studies ^3^,^12^,^16^-^18^.

In conclusion, with the limitation of this study, the body temperature did not affect the torsional strength of the experimental instruments manufactured from similar Blue and Gold technology. However, the Blue NiTi instruments presented significantly lower angular deflection than Gold instruments at 36 °C temperature.
